# Vaping danger: A hidden threat among Malaysia's youth

**DOI:** 10.51866/lte.847

**Published:** 2025-01-26

**Authors:** Norsiah Ali, Muhamad Hafiz Harun, Saravanan Mahendra, Masseni Abd Aziz

**Affiliations:** 1 MBBS, MMedFamMed, Peringgit Health Clinic, Jalan Pantai Peringgit, Melaka, Malaysia. Email: doktorhafiz@yahoo.com; 2 MD, MMedFamMed, Masjid Tanah Health Clinic, Jalan Besar Masjid Tanah, Alor Gajah, Melaka, Malaysia.; 3 MD, MMedFamMed, Seremban Health Clinic, Jalan Rasah, Seremban, Negeri Sembilan, Malaysia.; 4 MD, MMedFamMed, Durian Tunggal Health Clinic, Jalan Padang Keladi, Durian Tunggal, Alor Gajah, Melaka, Malaysia.

**Keywords:** Vaping, Drug, Mushroom

**Dear editor,**

Over the past few years, there has been a rising trend of young adult vapers presenting to health clinics across Malaysia with signs and symptoms such as dizziness, vomiting, eye redness, abnormal behaviours and seizures. Publishing this case aims to raise awareness among primary care doctors regarding the latest trends in substance use among Malaysian young adults and school-aged teenagers. Without prior experience in managing addiction cases or a high index of suspicion for substance use, such cases are at risk of being misdiagnosed and mistreated, especially since many new psychoactive substances (NPSs) cannot be detected with standard urine drug test kits.

For instance, the case of Mr K highlights the abovementioned issue. Mr K was a previously healthy 15-year-old student who was brought to a health clinic after being caught vaping with friends in a school toilet. He presented with drowsiness, one episode of vomiting, bilateral eye redness, unsteady gait and incoherent speech. However, there were no reports of shortness of breath, chest pain, palpitations, seizures, hallucinations, delusions or aggressive behaviours.

Upon evaluation, Mr K shared that peer pressure and curiosity led him to start vaping at the age of 15 years. Recently, he had been using a magic mushroom-flavoured vape, introduced by a close friend. He denied any history of drug or alcohol use and admitted to purchasing the vape from an unknown dealer via Telegram. Mr K expressed that he found the euphoria and relaxation from vaping to be a relief from painful childhood memories.

Mr K came from a low socioeconomic background and was raised by his grandparents. His father was incarcerated for drug-related offences, and his mother was working in Singapore. He reported feelings of loneliness and a lack of parental attention. At school, he struggled with disciplinary issues and poor academic performance.

Clinical assessment at the health clinic showed that he was alert, with a Glasgow Coma Scale score of 15/15, reactive pupils and coherent speech. His vital signs were stable: pulse rate, 55 beats/min; BP 113/60 mmHg; respiratory rate, 18 breaths/min; temperature, 36.9°C; and SpO2 level, 98% on room air. His cardiovascular and neurological findings were unremarkable, and a urine drug test revealed negative results. The diagnosis was non-e-cigarette or vaping product use-associated lung injury. He was referred to the hospital for further evaluation and monitoring.

At the hospital, Mr K’s symptoms resolved, and his vital signs remained stable. Electrocardiography showed sinus bradycardia (HR: 50 beats/min), with no acute ischaemic changes or prolonged QTc interval. Laboratory investigations, including full blood count, renal and liver profiles and blood gases, showed unremarkable findings. He was monitored for vape intoxication and discharged 24 hours later.

Due to a high index of suspicion for substance use, the school counsellor was contacted and managed to secure three vape devices, which were submitted to the Malaysian Chemistry Department for analysis. The results, received 2 weeks later, confirmed that the vape liquid contained MDMB-4en-PINACA, a synthetic cannabinoid classified under the Malaysian Dangerous Drugs Act 1952. No psilocybin compounds were detected.

Vaping and e-cigarette use among youth raise significant concerns, particularly due to the effects of high nicotine levels on the developing brain. During adolescence, the prefrontal cortex, which is key for attention, executive function, cognitive function and impulse control, is still developing. Although human studies on the impact of nicotine from e-cigarettes on adolescents’ brains are limited, research on traditional smoking indicates detrimental effects on cognitive processing and long-term behavioural and psychiatric issues.

Beyond direct health hazards, vaping might normalise smoking behaviours and serve as a gateway to conventional cigarette use.^1^ E-cigarettes may lead youth to develop nicotine dependence or become ‘dual users’ of both e-cigarettes and traditional cigarettes, increasing their overall nicotine consumption.^1,2^ Longitudinal studies link youth e-cigarette use to subsequent traditional cigarette use.^1^

A disturbing trend is seen among vape users. They move a step further, altering vaping devices by injecting or adding illegal substances into the vaping liquid. The fragrant vape juices make these dangerous and highly addictive drugs difficult to detect and can expose unknowing students to harmful and potentially deadly substances. One of the most popular or trending substances they use is magic mushroom flavour, which contains MDMB-4en-PINACA and produces a euphoric effect.^3^ It is a potent synthetic cannabinoid that has been used and sold online as a designer drug.^4^ The unique chemical structures frequently evade detection in standard drug screenings, complicating the identification of use and exposure.^5,6^ Synthetic cannabinoids are reported to be 100-200 times more potent than natural cannabinoids.^4^ They can cause sudden death due to arrhythmias, acute kidney injury and acute rhabdomyolysis.^4^

Nicotine liquid and gel have been excluded from the Poison Act (Amendment) 2022, leaving a regulatory gap that may endanger the public, particularly the youth. Malaysia needs a more comprehensive rule or act since the existing Control of Tobacco Product Regulations 2004 under the Food Act 1983 do not apply to vapes and other new smoking products. Without proper regulation and law enforcement, a vape or an e-cigarette is widely available to be purchased in shops, convenience stores and online stores. Some irresponsible sellers mix their vape juice with substances such as Amphetamine-type Stimulants (ATS), cannabis and NPS to attract consumers.

On 1 October 2024, Act 852 - the Control of Smoking Products for Public Health Act 2024 - was announced to be implemented.^7^ The Act was introduced to bring awareness and benefits to public health and environmental sustainability. It aims to curb smoking habits among the public and prevent the sale of smoking products to minors through law enforcement and continuous public education initiatives.

In summary, the unpredictable nature of vapes and NPSs, coupled with their potential for severe physiological and psychological effects, underscores the urgent need for increased awareness and research to mitigate the dangers of their usage. Implementation and enforcement of the new legislation regarding vaping are equally important to reduce harmful exposure to the young generation.

Primary care doctors should be highly vigilant in detecting this problem and able to offer assistance for quitting vaping.
